# Overestimation of maximal aerobic speed by the Université de Montréal track test and a 1500-m-time trial in soccer

**DOI:** 10.3389/fphys.2022.1023257

**Published:** 2022-10-11

**Authors:** Maximiliane Thron, Alexander Woll, Leon Klos, Sascha Härtel, Ludwig Ruf, Christian Kloss, Stefan Altmann

**Affiliations:** ^1^ Institute of Sports and Sports Science, Karlsruhe Institute of Technology, Karlsruhe, Germany; ^2^ TSG 1899 Hoffenheim, Zuzenhausen, Germany; ^3^ TSG ResearchLab GGmbH, Zuzenhausen, Germany

**Keywords:** football, fitness, endurance, field test, MAS, performance testing

## Abstract

**Introduction:** Maximal aerobic speed (MAS), usually measured by cardiopulmonary exercise testing (CPET) on a treadmill, is gaining popularity in soccer to determine aerobic performance. Several field tests are used to estimate MAS, although, gold standard methods are still not clarified. Therefore, this work aims 1) to compare two different CPET based methods to assess MAS and 2) to investigate the convergent validity of two common field tests to estimate MAS in soccer.

**Methods:** Thirteen trained male soccer players completed an CPET on a treadmill to determine two VO_2_-kinetic based definitions of MAS (MAS_Plateau_ = speed at onset of VO_2_-plateau = gold standard; MAS_30s_ = first speed of 30-s-interval of VO_2_max), the Université de Montreal Track Test (UMTT; V_UMTT_ = speed of the last stage), and a 1500-m-time trial (1500-m-TT; V_1500m_ = average speed). MAS_Plateau_, MAS_30s_, V_UMTT_, and V_1500m_ were compared using ANOVA. Additionally, limits of agreement analysis (LoA), Pearson’s r, and ICC were calculated between tests.

**Results:** MAS_30s_, V_UMTT,_ and V_1500m_ significantly overestimated MAS_Plateau_ by 0.99 km/h (ES = 1.61; *p* < 0.01), 1.61 km/h (ES = 2.03; *p* < 0.01) and 1.68 km/h (ES = 1.77; *p* < 0.01), respectively, with large LoA (-0.21 ≤ LoA≤3.55), however with large-to-very large correlations (0.65 ≤ r ≤ 0.87; *p* ≤ 0.02; 0.51 ≤ ICC≤ 0.85; *p* ≤ 0.03).

**Discussion:** The overestimation and large LoA of MAS_Plateau_ by all estimates indicate that 1) a uniform definition of MAS is needed and 2) the UMTT and a 1500-m-TT seem questionable for estimating MAS for trained soccer players on an individual basis, while regression equations might be suitable on a team level. The results of the present work contribute to the clarification of acquisition of MAS in soccer.

## Introduction

Given the high cardiorespiratory demands during a soccer match, in which professional players cover a distance up to 13 km, assessment of endurance performance with subsequent individual training prescription seems indispensable (Tanner and Gore, 2012; Altmann et al., 2020). Aerobic performance is often assessed by measuring the maximum oxygen uptake (VO_2_max). In soccer, VO_2_max is significantly related to the distance covered during a match and reflects aerobic capacity (Bangsbo et al., 1991; Aquino et al., 2020). The VO_2_max is commonly assessed by cardiopulmonary exercise testing (CPET) on a treadmill with incremental protocols (Kuipers et al., 2003; Riboli et al., 2021). A criterion for obtaining the true VO_2_max in such tests is the occurrence of a VO_2_-plateau which is defined by a lower increase in VO_2_ than 150 ml/min in at least the last minute of an incremental exercise (Meyer and Kindermann, 1999). Then, VO_2_max is defined as the maximal 30-s-interval of VO_2_ during an incremental CPET usually occurring at the termination of the test due to exhaustion. However, while being mainly an aerobic marker, VO_2_max is reached with efforts above the onset of the plateau associated with a higher input of anaerobic resources (Billat and Koralsztein, 1996). Therefore, the way VO_2_max is commonly measured, it incorporates not only aerobic but to some extent also anaerobic resources. Importantly, the anaerobic contribution can largely differ between athletes. This leads to different lengths of the VO_2_-plateau until exhaustion is reached, thereby distinguishing between athletes depending on their utilization of anaerobic resources. More specifically, athletes mainly relying on aerobic resources show a short VO_2_-plateau, whereby athletes using a greater amount of anaerobic resources display a longer plateau (Petot et al., 2012). As the velocity associated with the 30-s-interval of VO_2_max is commonly used for prescribing training intensities, it might trigger the aerobic and anaerobic energy pathways to different extents depending on the physiological profile of the respective athlete possibly leading to a non-optimal training adaptation. A parameter that addresses the mentioned shortcomings of VO_2_max is the maximal aerobic speed (MAS) which has recently gained popularity in scientific literature and practice. The MAS was firstly described by Di Prampero et al. (1986) as the minimum speed at which VO_2_-consumption stops increasing despite a further increase in load, i.e., the onset of the VO_2_-plateau, in the current study referred to as MAS_Plateau_. Based on MAS_Plateau_, training intensities aiming to address mainly aerobic resources to the same extent for different athletes can be set. CPET on a treadmill is used as a gold standard method to assess MAS. Nevertheless, different treadmill protocols and definitions of MAS exist, which also provide different results (Berthoin et al., 1996; Billat et al., 1996; Riboli et al., 2021). Besides MAS_Plateau_, the velocity of the 30-s-interval of VO_2_max (MAS_30s_) is often applied as an alternative method to assess MAS (Buchheit, 2008; Sandford et al., 2019). However, to date, no studies compared the currently most common definitions based on examination of VO_2_-kinetics, i.e., first velocity at onset of VO_2_-plateau (MAS_Plateau_) and the first velocity of 30-s-interval of VO_2_max (MAS_30s_).

Despite ambiguities in the gold standard method, simplified methods in the field are used to estimate MAS, such as incremental continuous field tests like Université de Montréal Track Test (UMTT) or different set time and distance trials (TT) (Léger and Boucher, 1980; Léger et al., 1988; Bangsbo et al., 2008; Clarke et al., 2016; Sandford et al., 2019). In terms of the validity of the UMTT, Berthoin et al. (1996) and Souza et al. (2014) could not find any significant differences between MAS and V_UMTT_, whereas Lacour et al. (1991) revealed an overestimation of MAS by V_UMTT_. Regarding set distance time trials to assess MAS, the current literature also reveals conflicting results. Set distance TT yielded similar results (Souza et al., 2014; Bellenger et al., 2015; Lundquist et al., 2021) or overestimated MAS and V_UMTT_ (Sandford et al., 2019; Darendeli et al., 2021). Due to contrary results in the current literature on field tests to estimate MAS, Buchheit (2010) proposed to distinguish the results obtained from CPET, from those obtained from field tests by designating the values obtained from CPET as MAS and the estimates from a field test as V_Test_. This reinforces the importance of reporting exact definitions and methods. Furthermore, most of the above-mentioned studies were conducted with runners and sports students, which does not allow for a clear conclusion about soccer.

To address these shortcomings, the aims of this study were 1) to compare two different VO2-kinetic based methods, i.e., first velocity at onset of VO_2_-plateau (MAS_Plateau_) and first velocity of 30-s-interval of VO_2_max (MAS_30s_), using CPET on a treadmill, and 2) to investigate convergent validity of both the UMTT and a 1500-m-TT in relation to a VO_2_-kinetic based MAS, i.e. MAS_Plateau_, in soccer. Thus, this work contributes to clarify the implementation of the gold standard method for assessing MAS in soccer and whether the assessment can be simplified by the UMTT or a 1500-m-TT.

## Materials and methods

### Study design

Thirteen trained male soccer players performed three tests at three different occasions, with a minimum of seven and a maximum of 21 days between the tests and a training rest of at least 24 h before each test. Tests were conducted in the same order for all participants: 1) UMTT on a 400-m-running track, 2) CPET on a treadmill, and 3) a 1500-m-TT on a 400-m-running track. During the incremental treadmill test, MAS_Plateau_ [km/h] and MAS_30s_ [km/h] were determined by examining the VO_2_-kinetics. Moreover, the main parameters measured during field testing were: V_UMTT_ [km/h], V_1500m_ [km/h], and V_calc_ [km/h]. MAS_30s_, V_UMTT_, V_1500m_, and V_calc_ were compared to MAS_Plateau_ which is considered as the gold standard method in the current study.

### Sample

The sample consisted of n = 13 trained male soccer players classified as tier two athletes (Mckay et al., 2022) (mean ± SD: age: 25.38 ± 2.75 years; height: 178.51 ± 7.82 cm; weight: 78.60 ± 8.85 kg; fat mass proportion: 16.86 ± 4.78%; VO_2_max: 49.92 ± 3.15 ml/kg/min; exercise frequency: 4.50 ± 0.50 times/week; soccer experience: 21.67 ± 3.40 years). Players’ health was checked and confirmed by the Physical Activity Readiness-Questionnaire (Tremblay et al., 2012). Each subject was informed about the study procedure and possible risks and agreed to participate by signing a consent form. As per the local legislation, this study was exempt from full ethics review by the institutional review board, due to this being an anonymous study containing anonymous data. To avoid bias, goal keepers were excluded from this study (Altmann et al., 2020).

### Variables and procedures

#### Université de Montréal track test

The UMTT was conducted on a 400-m-tartan track. Large cones were placed at 50-m-intervals, small cones after the first 33.33 m and at 100-m-intervals. The speed was controlled by an acoustic signal at which the cones had to be reached. The initial speed was set at 10 km/h and every 2 min the speed was increased by 1 km/h (Berthoin et al., 1996; Billat and Koralsztein, 1996; Bellenger et al., 2015; Darendeli et al., 2021). Each subject was equipped with a previously validated chest strap (H7 or H10, Polar Electro, Kempele, Finland) for monitoring HR (Speer et al., 2020; Hernández-Vicente et al., 2021). The test was completed as soon as the subject was no longer able to reach the specified cone at the acoustic signal. V_UMTT_ was determined as the speed of the last stage. If the last stage had not been finished, V_UMTT_ was calculated using the following formula: V_UMTT_ = speed of the last completed stage [km/h]+time in last stage [s]/120 s (Berthoin et al., 1996). In addition, the HRmax_UMTT_ and RPE_UMTT_ were recorded after the end of the test to assess physical exhaustion.

#### Cardiopulmonary exercise testing

To assess MAS_Plateau_ and MAS_30s_, CPET on a treadmill with ±0.1 km/h speed accuracy was performed (Woodway PPSmed 55 and PPSmed L70; WOODWAY GmbH; Weil am Rhein; Germany). The test protocol started at 6 km/h and increased every 3 min by 2 km/h with a treadmill incline of 1% to reflect the energy expenditure of outdoor running (Jones and Doust, 1996). Between each stage, the subject rested for 0.5 min and the test got terminated by the subject due to voluntary exhaustion (Dickhuth and Badtke, 2007). Breath-by-breath ventilatory data were obtained using the Metalyzer 3B spirometer and the appropriate MetaSoft three software (Cortex Biophysik GmbH; Leipzig; Germany) with which the data was prepared as 15-s moving average values for further analysis. This technology enables accurate and precise determination of the individual VO_2_-kinetics (Vogler et al., 2010). Gas sensors were calibrated using gases of known concentrations (15% O_2_, 5% CO_2_), and the turbine volume transducer was calibrated using a 3-l syringe (Cortex Biophysik GmbH; Leipzig; Germany).

The VO_2_-data were first examined for a plateau which is defined as a lower increase in VO_2_ than 150 ml/min in at least the last minute of exercise (Meyer and Kindermann, 1999). The MAS_Plateau_ represents the first velocity when reaching this plateau (Billat and Koralsztein, 1996). Additionally, the velocity at the onset of the 30-s-interval of VO_2_max (MAS_30s_) was determined, as this is another common definition for the determination of MAS (Buchheit, 2008; Sandford et al., 2019). The VO_2_max was defined as the highest 30-s-interval of VO_2_. The RER_end_ represented the highest value of the quotient VCO_2_/VO_2_ during the end of exercise. The HRmax was assessed using a chest strap (H7 or H10; Polar Electro; Kempele; Finland). In addition, RPE was queried after test termination. Immediately after the test, 20 μl of capillary blood was collected from the right earlobe and analyzed by the BIOSEN C-Line lactate analyzer (EKF Diagnostic; Barleben; Germany) to assess the maximal lactate value reached during the treadmill test (La_end_). In order to achieve physical exhaustion and thus VO_2_max, the collected data were analyzed for the following exercise criteria, of which at least two had to be fulfilled (Neumann, 2013): RER_end_ ≥ 1.0 (Dickhuth and Kindermann, 2002); RPE ≥17 (Sangan et al., 2021); HRmax ≥210-age, La_end_ ≥ 8 mmol/L, and reaching a VO_2_-plateau (Marées and Heck, 2003).

#### 1500-m-time trial

To ensure reliability of the 1500-m-TT, subjects performed a habituation session 1 week in advance (Clarke et al., 2016). For HR measurement, each subject received a chest strap (H7 or H10; Polar Electro; Kempele; Finland). The subjects ran on a 400-m tartan track and performed a warm-up (at least 400 m jogging; 100 m easy acceleration runs; 3 min stretching) right before the start of the TT. During the TT, subjects were instructed to keep their speed as even as possible. The time to complete the 1,500 m was measured with a stopwatch, from which the average speed in km/h (V_1500m_) was determined (Darendeli et al., 2021). In addition, the regression equation of Bellenger et al. (2015) was used to calculate an approximation to the true MAS (V_calc_ = V_1500m_*(0.766 + 0.117*1.5 km)). HRmax_1500m_ and RPE_1500m_ were recorded after test termination to ensure physical exhaustion.

### Statistical analysis

Statistical analysis and creation of graphics were carried out with the statistical software IBM SPSS^©^ Statistics (version 27). To detect possible differences between the parameters MAS_Plateau_/V_UMTT_/V_1500m_/V_calc_, HRmax/HRmax_UMTT_/HRmax_1500m_, and RPE/RPE_UMTT_/RPE_1500m_, ANOVA with repeated measures and Bonferroni post-hoc tests were calculated. In addition, t-tests for dependent samples were used for the comparison of MAS_Plateau_ vs. MAS_30s_, and MAS_Plateau_ vs. V_calc_. The effect sizes of the ANOVA results were estimated by partial eta^2^: 0.01≤η_p_^2^<0.06 is considered a small, 0.06≤η_p_^2^<0.14 as a medium, and 0.14≥η_p_^2^ as a large effect (Cohen, 1988). The effect sizes (ES) of the t-tests and post-hoc tests were calculated using Cohen’s d: 0.2 ≤ ES < 0.5 represent small, 0.5 ≤ ES < 0.8 medium, and ES ≥ 0.8 represent large effects (Cohen, 1988). Absolute agreement between MAS_Plateau_, V_UMTT_, and V_1500m_ and between MAS_Plateau_ and MAS_30s_, and MAS_Plateau_ and V_calc_ was determined with the limits-of-agreement analysis (LoA analysis) and Bland-Altman plots (Altman and Bland, 1983). In addition, Pearson’s correlation coefficient r and the intraclass correlation coefficient (ICC 3.1) were calculated. According to Hopkins (2004), the magnitude of the correlation was considered to be small (0.1 ≤ r/ICC<0.3), medium (0.3 ≤ r/ICC<0.5), large (0.5 ≤ r/ICC<0.7), very large (0.7 ≤ r/ICC<0.9), and almost perfect (r/ICC≥0.9) classifications. The significance level for all calculations was set at *p* < 0.05.

## Results

**FIGURE 1:**
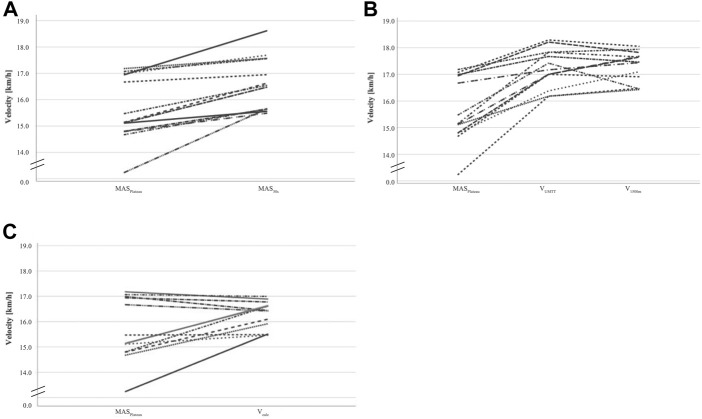
Spaghetti plots for intra- and interindividual comparison of **(A)** MAS_Plateau_ and MAS_30s_; **(B)** MAS_Plateau_, V_UMTT_, and V_1500m_; and **(C)** MAS_Plateau_ and V_calc_.

Each subject met three or more of the specified criteria, so that physical exhaustion was ensured. The mean values and standard deviations of the recorded parameters for the incremental treadmill test, UMTT and the 1500-m-TT are shown in Table 1. Moreover, individual progressions of MAS_Plateau_/MAS_30s_, MAS_Plateau_/V_UMTT_/V_1500m_, and MAS_Plateau_/V_calc_ are visualized as spaghetti plots in Figure 1. These descriptive results already indicate an overestimation of MAS_Plateau_ by the estimations, i.e., MAS_30s_, V_UMTT_, V_1500m_, and V_calc_.

**TABLE 1:** Descriptive results for the CPET on the treadmill, UMTT, and 1500-m-TT. Results are presented as mean values ±SD.

	MAS/v_Test_ [km/h]	RPE [N/A]	HR_max_ [bpm]	VO_2_max [ml/kg/min]	La_end_ [mmol/l]	RER_end_ [N/A]
	mean ± SD					
*CPET*	MAS_Plateau_	19.38 ± 0.77	185.54 ± 7.67	49.92 ± 3.15	8.52 ± 2.81	1.18 ± 0.05
	15.63 ± 1.22					
	MAS_30s_					
	16.62 ± 1.01					
	V_max_					
	17.29 ± 1.07					
*UMTT*	V_UMTT:_17.24 ± 0.71	17.85 ± 1.21	186.85 ± 7.06	-	-	-
*1500-m-TT*	V_1500m_	17.77 ± 1.74	183.54 ± 6.65	-	-	-
	17.31 ± 0.58					
	V_calc_					
	16.30 ± 0.54					

SD, standard deviation; CPET, cardiopulmonary exercise testing; UMTT, Université de Montréal Track Test; 1500-m-TT, 1500-m-time trial; MAS, maximal aerobic speed; V_Test_, estimate of MAS by field tests; MAS_Plateau_, maximal aerobic speed assessed as velocity of onset of VO_2_-plateau; MAS_30s_, first velocity of 30-s-interval of VO_2_max; V_max_, maximal speed achieved during CPET; V_UMTT_, maximal velocity in UMTT; V_1500m_, average velocity in 1500-m-TT; V_calc_, velocity estimated by regression equation with final speed of 1500-m-TT (V_1500m_*(0.766 + 0.117*1.5 km); Bellenger et al., 2015); RPE, ratings of perceived exertion; HR_max_, maximal heart rate; VO_2_max, maximal oxygen update; La_end_, lactate value at test termination; RER_end_, respiratory exchange ratio at test termination.

**FIGURE 2:**
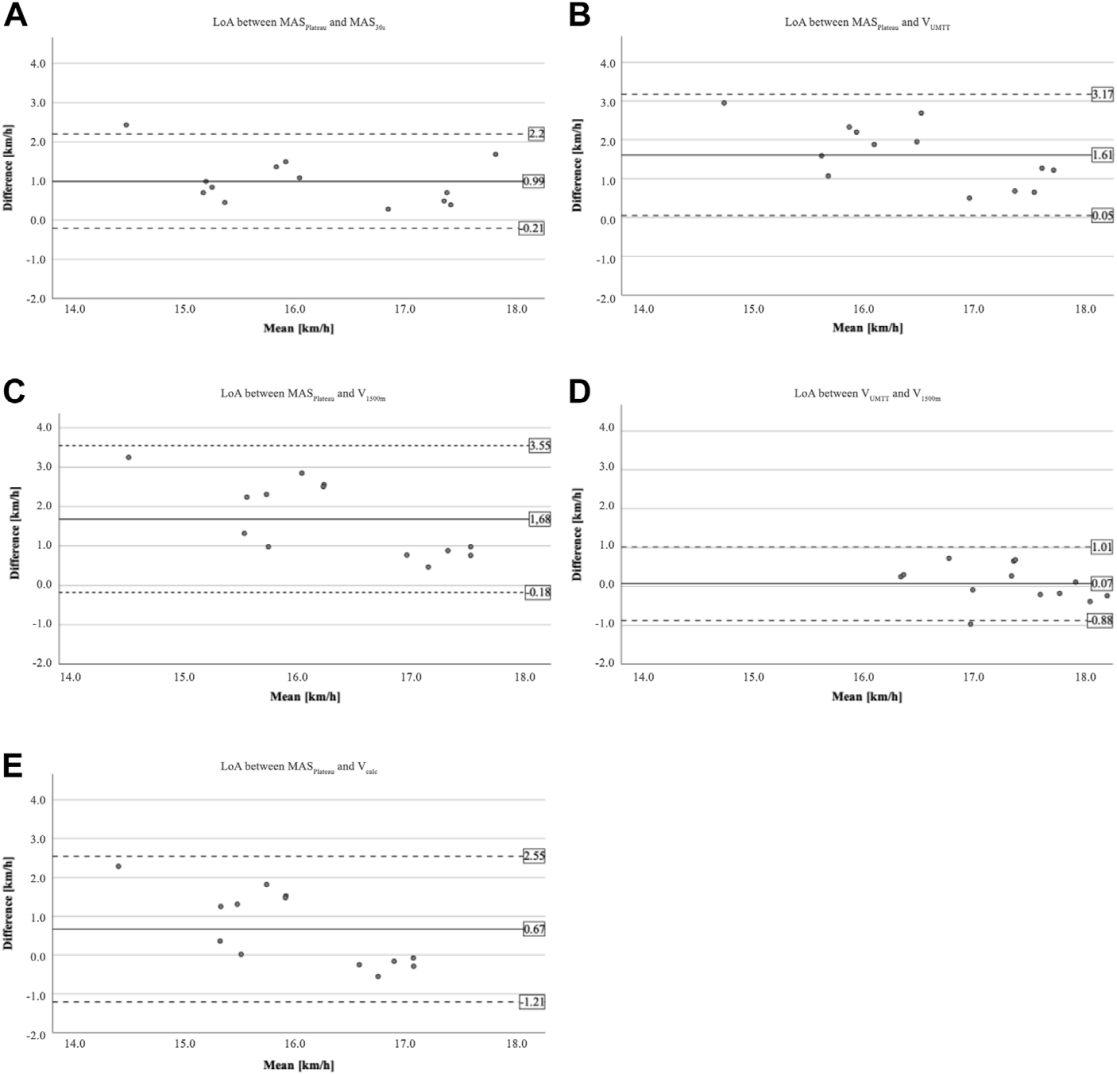
Bland-Altman plots for limits-of-agreement analysis (LoA analysis) between **(A)** MAS_Plateau_ and MAS_30s_; **(B)** MAS_Plateau_ and V_UMTT_; **(C)** MAS_Plateau_ andV_1500m_; **(D)** V_UMTT_ and V_1500m_; and **(E)** MAS_Plateau_ and V_calc_. The solid lines represent the mean difference, the dashed lines represent the limits of agreement (±1.96 SD).

The comparison of both VO_2_-kinetic based methods to assess MAS resulting in the parameters MAS_Plateau_ and MAS_30s_ (see Table 2) shows a significant increase by 0.99 km/h (*p* < 0.01) with a large effect (ES = 1.61) and Limits of Agreement (LoA) ranging from -0.21–2.20 km/h (see Figure 2). Though, results of correlation analysis show very large correlations between MAS_Plateau_ and MAS_30s_ (r = 0.87; ICC = 0.85; see Table 2).

**TABLE 2:** Results of ANOVA, Bonferroni post-hoc tests, t-tests, and correlation analysis.

	MD (95%CI)	p	*n*_p_^2^	ES (95%CI)	r (95%CI)	p	ICC (95%CI)	p
*MAS*_*Plateau*_ */ MAS*_*30s*_	0.99 km/h (0.62–1.36)	<0.01*	-	1.61 (0.76–2.43)	0.87 (0.60–0.96)	<0.01*	0.85 (0.58–0.95)	<0.01*
*MAS*_*Plateau*_ */ V*_*calc*_	0.67 km/h (0.09–1.25)	0.03*	-	0.70 (0.07–1.25)	0.65 (0.16–0.90)	0.02*	0.40 (-0.08–0.80)	0.04*
*MAS*_*Plateau*_ */ V*_*UMTT*_ */ V*_*1500m*_	-	<0.01*	0.77	-	-	-	0.62 (0.31–0.85)	<0.01*
*MAS*_*Plateau*_ */ V*_*UMTT*_	1.61 km/h (1.00–2.23)	<0.01*	-	2.03 (1.05–2.98)	0.79 (0.42–0.93)	<0.01*	0.69 (0.24–0.89)	<0.01*
*MAS*_*Plateau*_ */ V*_*1500m*_	1.68 km/h (0.95–2.42)	<0.01*	-	1.77 (0.87–2.64)	0.65 (0.16–0.90)	0.02*	0.51 (-0.40–0.82)	0.03*
*V*_*UMTT*_ */ V*_*1500m*_	0.07 km/h (-0.30–0.44)	0.99	-	0.14 (-0.41–0.69)	0.74 (0.32–0.92)	<0.01*	0.72 (0.31–0.91)	<0.01*
*HRmax/ HRmax*_*UMTT*_ */ HRmax*_*1500m*_	-	0.09	0.18	-	-	-	0.73 (0.47–0.90)	<0.01*
*HRmax/ HRmax*_*UMTT*_	1.31 bpm (-1.83–4.45)	0.81	-	0.31 (-0.24–0.87)	0.85 (0.56–0.95)	<0.01*	0.85 (0.58–0.95)	<0.01*
*HRmax/ HRmax*_*1500m*_	-2.00 bpm (-6.77–2.77)	0.80	-	0.32 (-0.24–0.88)	0.63 (0.13–0.88)	0.02*	0.63 (0.14–0.87)	<0.01*
*HRmax*_*UMTT*_ */ HRmax*_*1500m*_	-3.31 bpm (-7.34–0.73)	0.13	-	0.63 (0.02–1.22)	0.71 (0.27–0.91)	<0.01*	0.71 (0.29–0.90)	<0.01*
*RPE/ RPE*_*UMTT*_ */ RPE*_*1500m*_	-	<0.01*	0.47	-	-	-	0.40 (0.06–0.73)	0.01*
*RPE/ RPE*_*UMTT*_	-1.54 (-2.56–0.51)	<0.01*	-	1.16 (0.43–1.85)	0.16 (-0.43–0.65)	0.61	0.14 (-0.42–0.63)	0.31
*RPE/ RPE*_*1500m*_	-1.62 (-2.73–0.50)	<0.01*	-	1.12 (0.40–1.80)	0.57 (0.03–0.85)	0.04*	0.42 (-0.14–0.78)	0.07
*RPE*_*UMTT*_ */ RPE*_*1500m*_	-0.08 (-1.23–1.08)	>0.99	-	0.05 (-0.49–0.59)	0.53 (-0.02–0.84)	0.06	0.51 (-0.04–0.82)	0.03*

MD, mean difference; 95% CI, 95% Confidence Interval; p, significance level; *—*p* < 0.05, i.e., significant difference; η_p_^2^, partial squared eta; ES, effect size of Bonferroni post-hoc tests and t-tests; r, Pearson’s Coefficient of Correlation; ICC, Intra Class Correlation Coefficient; MAS_Plateau_, maximal aerobic speed assessed as velocity of onset of VO_2_-plateau; MAS_30s_, first velocity of 30-s-interval of VO_2_max; V_UMTT_, maximal velocity in UMTT; V_1500m_, average velocity in 1500-m-TT; V_calc_, velocity estimated by regression equation with final speed of 1500-m-TT (V_1500m_*(0.766 + 0.117*1.5 km); Bellenger et al., 2015); RPE, ratings of perceived exertion; HR_max_, maximal heart rate.

Moreover, V_UMTT_ and V_1500m_ overestimate MAS_Plateau_ by 1.61 km/h (*p* < 0.01; ES = 2.03) and 1.68 km/h (*p* < 0.01; ES = 1.77), respectively. Between V_UMTT_ and V_1500m_, no significant difference (*p* = 0.99; ES = 0.14) was found. The LoA between V_UMTT_ and V_1500m_ range from -0.8–1.01 km/h, between MAS_Plateau_ and V_UMTT_ from 0.05 to 3.17 km/h, and between MAS_Plateau_ and V_1500m_ from -0.18–3.55 km/h. The correlations between the three velocities are large to very large (0.65 ≤ r ≤ 0.79; *p* ≤ 0.02; 0.51 ≤ ICC≤0.72; *p* ≤ 0.03). The comparison of MAS_Plateau_ and V_calc_ shows a significant difference (MD = 0.67 km/h; *p* = 0.03; ES = 0.70). In addition, the LoA between MAS_Plateau_ and V_calc_ range from -1.21–2.55 km/h. There is a medium to large correlation between MAS_Plateau_ and V_calc_ (r = 0.65; *p* = 0.02; ICC = 0.40; *p* = 0.04).

## Discussion

### Main findings

The first aim of this study was to compare two different VO_2_-kinetic based methods to determine MAS in soccer players, i.e., MAS_Plateau_ and MAS_30s_, using CPET on a treadmill. MAS_Plateau_ was consistently overestimated by MAS_30s_, nevertheless, MAS_Plateau_ and MAS_30s_ were highly correlated.

The second aim was to investigate the convergent validity of the UMTT and a 1500-m-TT to estimate MAS_Plateau_. MAS_Plateau_ was overestimated by both V_UMTT_ and V_1500m_, nonetheless, large to very large correlations with MAS_Plateau_ were found. Further, the calculated speed from the 1500-m-TT according to Bellenger et al. (2015), V_calc_, was significantly higher than MAS_Plateau_.

### Discussion of MAS assessment *via* CPET

The results of the CPET to determine MAS illustrate the difference between two of the common definitions of MAS. Since VO_2_max in healthy subjects almost always occurs before the end of an physical exhaustion exercise and a VO_2_-plateau by definition lasts at least 1 minute (Meyer and Kindermann, 1999), a significant difference between MAS_Plateau_ and MAS_30s_ was expected. The mean difference of 0.99 km/h indicates that after reaching MAS_Plateau_, subjects maintained their performance for an average of almost 1.5 min with a higher proportion of anaerobic resources until MAS_30s_ was reached (Buchheit, 2010). Because MAS_Plateau_ represents the first velocity when reaching a VO_2_-plateau (Di Prampero et al., 1986) and therefore reflects the maximal speed with mainly aerobic resources, efforts above the onset of the plateau are associated with a higher input of anaerobic resources. Hence, velocities above the onset of a VO_2_-plateau should not be attributed as a MAS. The very large effect size illustrates the practical relevance of this difference and the very large correlation between the two velocities supports the consistent overestimation of MAS_Plateau_. Therefore, MAS should be determined individually based on VO_2_-kinetics by investigating the plateau. In addition, these results suggest that studies that used V_max_ (Berthoin et al., 1996) or MAS_30s_ (Sandford et al., 2019) to validate field testing procedures, probably did not use the “true” MAS determined by CPET with examining the onset of the VO_2_-plateau as a gold standard, which may have biased the results.

### Discussion of UMTT and 1500-m-TT

The LoA between MAS_Plateau_ and V_UMTT_ indicate a wide dispersion of individual differences. Moreover, the significant overestimation found in this study is similar to the results of Lacour et al. (1991) who also reported a higher V_UMTT_ than MAS (+0.25 ± 0.07 km/h, *p* = 0.03) and a nearly perfect correlation (r = 0.92; *p* < 0.01) in a group of runners despite using a different protocol and a different definition for MAS. In contrast, Berthoin et al. (1996) and Souza et al. (2014) did not find differences between MAS and V_UMTT_, but also used different protocols and definitions of MAS. Berthoin et al. (1996) determined the velocity of the last stage for both MAS (assessed by CPET on a treadmill) and V_UMTT_, which means that they did not distinguish between MAS and V_max_. On the contrary, in the present study, V_UMTT_ was compared to MAS_Plateau_, which represents the first velocity of the VO_2_-plateau. The UMTT represents an incremental test similar to CPET on a treadmill and because the final speed achieved during the UMTT is used as V_UMTT_ the significant difference to MAS_Plateau_ assessed as the onset of a VO_2_-plateau can be explained.

Additionally, V_1500m_ shows a systematic overestimation of MAS_Plateau_. Nevertheless, both the relatively large LoA and the large 95% CI of the mean difference indicate interindividual discrepancies in the differences between MAS_Plateau_ and V_1500m_. Sandford et al. (2019) also detected an overestimation of MAS by V_1500m_. The overestimation could be explained by the assumption that the distance for the total sample may have been too short to include only aerobic resources, but additionally anaerobic resources to run the 1,500 m in the best possible time. The fact that for some players the V_1500m_ was considerably closer to the MAS_Plateau_ than for others could indicate a heterogeneous character of the endurance performance of the sample. This may be due to the non-professional level of the sample on the one hand and on the other hand to position-specific differences. Different endurance profiles related to the player position could already be demonstrated in part (Altmann et al., 2020). Therefore, different distances should be used when implementing TT to estimate MAS_Plateau_ depending on the endurance performance level.

To address this issue, Bellenger et al. (2015) propose a regression equation (V_calc_) to estimate MAS by set distance TT with distances between 1,600 m and 2,200 m. The comparison of MAS_Plateau_ and V_calc_ shows similar results as the comparison of MAS_Plateau_ and V_1500m_. In this sense, V_calc_ also overestimates MAS_Plateau_. If individual variations are considered, it is plausible that, as with V_1500m,_ the dispersion around the mean difference is very large. This was to be expected, since V_calc_ is calculated from V_1500m_. The individual differences were merely shifted downward, so that they scatter both to the positive and to the negative. However, it should be noted that the regression equation used to calculate V_calc_ was set up by Bellenger et al. (2015) using V_UMTT_ as reference. This could additionally explain the overestimation of the MAS_Plateau_ in this study.

Moreover, the comparison of the two velocities V_1500m_ and V_UMTT_ achieved during the field test have the lowest mean difference and narrowest range of variation, as well as a very large correlation. Both Bellenger et al. (2015) and Lundquist et al. (2021) found high to very high levels of agreement between V_1400m_–V_2000m_ and V_UMTT_ in male and female Australian Rules Football players. This also indicates a similar intensity of the two test procedures for team sports athletes.

### Delimitations and limitations

The gold standard method to assess MAS has not yet been clarified, in particular it is unclear which treadmill protocol leads to the “true” MAS, especially in soccer. A major delimitation of our study is that we used a protocol with a 0.5 min break between the stages, which commonly serves to measure lactate *via* capillary blood between the stages of an incremental treadmill test. When analysing the individual VO_2_-kinetics, we determined that the break between the stages is a major limitation. This pause probably allowed a short recovery, so that VO_2_ progression delayed, especially at higher speeds at the beginning of a stage. This influences individual determination of MAS based on the VO_2_-kinetics and possibly postpones physical exhaustion and thus reaching VO_2_max (Metaxas et al., 2005). As a further delimitation, different environmental conditions may have influenced performance during the different test procedures. For example, some players completed the incremental treadmill test in the morning or at noon, with the UMTT and 1500-m-TT occurring in the evening for all subjects. Changes in performance at different times of the day have already been confirmed (Chtourou and Souissi, 2012). A limitation of our study is that the field tests were performed in different groups while the treadmill test was performed individually. This might have induced different levels of motivation in the subjects at the test time points. A last point to consider is the relatively heterogeneous aerobic endurance performance of the subjects. The MAS_Plateau_ of the sample shows a range of 13.23–17.18 km/h. Due to heterogeneous performance, the use of a TT with the same distance for all subjects is questionable. However, a habituation session was performed for improvement of independent speed control during the 1500-m-TT.

### Future research

In order to interpret and compare different study results, it is important to use a consistent gold standard method for the assessment of MAS. Since different definitions are used to determine MAS, future research is necessary to clearly delineate MAS_Plateau_, MAS_30s_ and V_max_. Because previous studies on the validity of field test procedures have not been conducted with a uniform gold standard method and because MAS determination *via* an incremental treadmill test claims many resources, further investigation of the validity of existing field test procedures or the development of new test procedures is essential. Especially in team sports and its divergent endurance performance levels, there is a great potential for research in this regard. Regarding a TT, e.g., a classification of players into different endurance profiles based on norm values of MAS or by different expressions of anaerobic speed reserve (Sandford et al., 2021) and the assignment of these levels to appropriate distances to estimate MAS should be investigated in the future. Thus, a more accurate estimate of MAS using a TT would be possible. Though, the sample consisted of trained soccer players, the results can not directly be transferred to professional soccer. Therefore, more research regarding the assessment of MAS is required with professional soccer players.

In addition, technological evolutions may simplify MAS estimation in the future. On the one hand, global positioning systems could make it easier and presumably more accurate to record velocities in field tests, and on the other hand, algorithms could be developed and validated that estimate the current MAS based on certain physiological parameters - at best during training - and indicate any acute adjustments (Leser et al., 2011). Given the tight schedules of soccer players and coaches, integrating physiological diagnostics into the usual training could lead to a more efficient time schedules of players and coaches.

### Practical applications

The UMTT and 1500-m-TT do not appear to be valid and thus less appropriate methods for estimating MAS for male soccer players. The LoA for both, UMTT and 1500-m-TT, indicate the deviations from MAS are higher than typical improvements of MAS achieved through specific training programs (Denadai et al., 2006; González-Mohíno et al., 2016). Therefore, training based on MAS estimations *via* UMTT or a TT might lead to much higher intensities than expected when using the true MAS, probably resulting in less improvements in performance, overload, or even a higher risk of injuries. For this purpose, it is recommended to either set up regression equations using a consistent gold standard method for estimating MAS using the V_UMTT_ or V_1500m_ or to choose individual distances for TT adjusted to the endurance performance level. Moreover, adapted training programs are possible, when the individual intensity of V_UMTT_ or V_1500m_ in deviation to MAS is known.

## Conclusion

To conclude, our results indicate that different definitions of MAS lead to different results and that estimates by field tests are non-appropriate methods to determine MAS in trained soccer players. Conversely, using such methods may result in researchers and practitioners using too high intensities. This indicates the necessity of MAS-assessment by investigating VO_2_-kinetics and examining the onset of the VO_2_-plateau or the use of adjusted or new field tests to estimate MAS. This work provides the first evidence on the validity of MAS estimation of different field tests in soccer players. To improve MAS estimation in field tests, further valid and time saving methods as well as practical instructions for coaches should be developed in soccer in the future.

## Data Availability

The raw data supporting the conclusions of this article will be made available by the authors, without undue reservation.
